# Linking moss structural and functional traits to soil water fluxes and soil erosion

**DOI:** 10.1093/aobpla/plag011

**Published:** 2026-02-18

**Authors:** Corinna Gall, Anne Beschorner, Julia Ehmann, Philipp Gries, Martin Nebel, Thomas Scholten, Steffen Seitz

**Affiliations:** Soil Science and Geomorphology, Department of Geosciences, University of Tübingen, Rümelinstr. 19-23, Tübingen 72070, Germany; Soil Science and Geomorphology, Department of Geosciences, University of Tübingen, Rümelinstr. 19-23, Tübingen 72070, Germany; Soil Science and Geomorphology, Department of Geosciences, University of Tübingen, Rümelinstr. 19-23, Tübingen 72070, Germany; Soil Science and Geomorphology, Department of Geosciences, University of Tübingen, Rümelinstr. 19-23, Tübingen 72070, Germany; Nees Institute for Biodiversity of Plants, University of Bonn, Meckenheimer Allee 170, Bonn 53115, Germany; Soil Science and Geomorphology, Department of Geosciences, University of Tübingen, Rümelinstr. 19-23, Tübingen 72070, Germany; Soil Science and Geomorphology, Department of Geosciences, University of Tübingen, Rümelinstr. 19-23, Tübingen 72070, Germany; Physical Geography, Institute of Geography, University of Osnabrück, Seminarstrasse 19 a/b, Osnabrück 49074, Germany

**Keywords:** bryophytes, rainfall simulation, ecohydrology, moss structural traits, water storage capacity, temperate forests, soil-water interactions

## Abstract

Mosses contribute to a multitude of ecosystem functions, and their structural and functional traits, such as shoot density or water storage capacity, can play an important role in performing these functions. It is widely known that mosses substantially reduce surface runoff and soil erosion, so the impact of mosses on soil hydrology is expected to be important worldwide. Nevertheless, it is poorly understood how moss species with different traits affect soil erosion and soil water fluxes. Therefore, this study aimed to assess the impact of the two moss species, *Thuidium tamariscinum* and *Rhytidiadelphus loreus*, on soil erosion, surface runoff, and water percolation and to investigate the influence of their structural traits. We conducted ex situ rainfall simulations with infiltration boxes that contain undisturbed topsoil samples. The two moss species differed significantly in their structural traits, with *R. loreus* showing longer shoots and branches, greater cushion height, and larger leaf area, while *T. tamariscinum* had a higher number of branches. Surprisingly, their water storage capacities were similar despite these morphological differences, suggesting a possible interplay of morphological features and trait resemblances, though the underlying mechanisms require further study. *R. loreus* and *T. tamariscinum* exhibited a comparable mitigating effect on sediment discharge and surface runoff, while moss cover had no significant influence on water percolation, regardless of species. Shoot density significantly correlates with reduced surface runoff, highlighting the key role of moss colony structure in hydrological processes.

## Teaser

This study highlights that mosses play an important role in reducing soil erosion and surface runoff, which is related to specific moss structural traits. Using rainfall simulations with infiltration boxes containing undisturbed topsoil, we compared three treatments: bare soil and soil covered by Rhytidiadelphus loreus or Thuidium tamariscinum. Despite pronounced differences in shoot and branch length, cushion height, leaf area, and number of branches, T. tamariscinum and R. loreus showed similar water storage capacities and comparable mitigation of surface runoff and sediment discharge, without a significant impact on water percolation. The ramification index emerged as a key trait, linking increased shoot branching to higher surface runoff and sediment discharge.

## Introduction

Mosses colonize a large part of Earth's surface in a variety of different ecosystems, ranging from arctic and boreal ecosystems to temperate and tropical forests, drylands, and even deserts (Hedenäs 2007, Lindo and Gonzalez 2010). In these ecosystems, mosses contribute to a multitude of functions related to carbon sequestration, nutrient cycling, organic matter decomposition, and plant pathogen control (Eldridge et al. 2023). Additionally, Porada et al. (2018) demonstrated that non-vascular vegetation, including mosses, plays a significant role in the global water cycle when represented in a process-based numerical model. For example, rainfall interception increases globally by 6.5 times on average, while mean annual evaporation rises by 61% with non-vascular vegetation (Porada et al. 2018). To illustrate these processes in simple terms, the hydrological balance within an ecosystem depends on the amount of rainfall that either runs off the surface, infiltrates into the soil, or is temporarily detained on or within the vegetation layer. In this context, detention refers to the temporary storage of water on the surface or within moss layers before it either evaporates or infiltrates into the soil. By increasing rainfall interception and detention, mosses reduce immediate surface runoff and promote greater water retention within the ecosystem in proportion to their dominance and abundance (Price et al. 1997, Slate et al. 2024).

In many forest ecosystems, mosses form an extensive, continuous, and thick ground cover, and they are known for their ability to absorb huge amounts of water, with some *Sphagnum* species even reaching over 5000% of their dry weight when saturated (Wang and Bader 2018). In Canadian boreal forests, moss layers have been shown to retain up to 16.8 mm of water, corresponding to approximately 21% of the precipitation input (Price et al. 1997). This also results in a higher soil moisture under mosses than without mosses (Siwach et al. 2021, Sun et al. 2021), whereby mosses with a higher water storage capacity also have a more positive influence on soil moisture (Oishi 2018, Hu et al. 2023). However, Thielen et al. (2021) were able to show that mosses do not reach their maximum water storage capacity during intense simulated rainfall, but instead release part of the water to the soil substrate, indicating that mosses do not necessarily act as an infiltration barrier under high rainfall intensity. Moreover, mosses considerably reduce surface runoff; for instance, Parsakhoo et al. (2012) reported a reduction of 67% on moss-covered forest road cutslopes compared to bare soil, while Gall et al. (2025a) observed an even greater reduction of 91% in moss-covered soil flumes. By decreasing surface runoff and providing a physical barrier against splash erosion, mosses generally mitigate soil erosion (Gall et al. 2022, 2025b). Through rainfall interception and increasing detention, mosses also lower the velocity of surface runoff (Juan et al. 2023), which further helps to prevent the detachment and transport of soil particles (Liu et al. 2016, Jing et al. 2025, Zeng et al. 2025).

The variation in species-specific water storage capacity among mosses is considerable (Wang and Bader 2018, Thielen et al. 2021, Volkova et al. 2023). Proctor et al. (1998) studied 16 moss species and reported values ranging from 108% to 2070% of their dry weight, highlighting substantial interspecific differences. Since this detention capacity represents a key component in the hydrological balance within an ecosystem, it is reasonable to assume that different moss species also exert different influences on other process components. There are only a few studies that investigate such species-specific effects on hydrological processes, and they come to different conclusions. While Tu et al. (2022) observed species-specific differences in surface runoff and infiltration rate in a soil flume experiment with rainfall simulations, Gall et al. (2025a) found no differences in surface runoff, water percolation, or sediment discharge between moss species in a similar experimental setup. This discrepancy may be due to the species selection, as the study by Tu et al. (2022) included a *Sphagnum* species alongside with two others, while Gall et al. (2025a) focused on structurally more similar moss species from temperate forests.

Structural traits of mosses are generally important for their ability to provide ecosystem functions, particularly in regulating hydrological processes (Eldridge and Rosentreter 1999). Their water absorption, for example, primarily occurs through external capillaries (ectohydric movement) formed by spaces between shoots, leaves, stems, and rhizoids, as well as specialized capillary structures like leaf bases and grooves (Proctor et al. 1998, Glime 2021). Structural traits such as leaf shape, arrangement, surface ornamentation, branch arrangement, and the presence of rhizoids or paraphyllia influence these capillary spaces (Schofield 1981). For example, several studies have shown that the water storage capacity of various subarctic moss species was significantly higher immediately after immersion, when water is still adhering to the leaf surface, than after drying the moss shoots on blotter paper for 2 minutes to remove the external water (Elumeeva et al. 2011, Volkova et al. 2023). However, Volkova et al. (2023) found that the measured standard deviations in water storage capacity with external water were also significantly higher than those without external water, indicating substantial variability in structural traits within one species, which demonstrates the complexity of the relationships.

An important step towards a better understanding of moss water storage is to directly link their structural traits to their water storage capacity. In this context, Thielen et al. (2021) discovered that the maximum water storage capacity of various mosses of temperate forests was increased in particular by small leaf areas and high leaf frequencies. Bengtsson et al. (2020) also highlighted the importance of leaf geometry for maximum water storage capacity, showing that leaf width, in particular, had a positive correlation, which was also true for leaf area, in contrast to the findings of Thielen et al. (2021). Recently, however, the study by Liu et al. (2024) found no significant correlation between leaf area and maximum water storage capacity for subtropical montane mosses. Nevertheless, it is widely recognized that there is a clear connection between the life form of mosses, which is the form of individual moss shoots growing together (Mägdefrau 1982, Bates 1998), and their ability to store water, which has been proven by several studies (Elumeeva et al. 2011, Oishi 2018, Lett et al. 2022).

While the hydrological importance of mosses at larger scales is increasingly recognized (Porada et al. 2018, Eldridge et al. 2023), the underlying structural traits that control their influence on soil water fluxes and soil erosion remain enigmatic. Although moss functional trait ecology is a rapidly developing field of science, trait-based studies related to water storage and soil properties are still scarce (Coe et al. 2024), and moss traits remain under-represented compared to those of vascular plants (Deilmann et al. 2024), requiring further in-depth investigation.

This study aimed to assess the impact of two moss species and their structural and functional traits on soil water fluxes and soil erosion compared to bare soil. Here, soil water fluxes are represented by surface runoff and water percolation, while soil erosion is expressed by sediment discharge. We used an experimental design that consists of an ex situ rainfall simulation with infiltration boxes that contain undisturbed topsoil samples of the same parent material. To test these species-specific effects, the following two hypotheses are formulated: (i) the two studied moss species differ in their structural traits, resulting in a difference in water storage capacity as a functional trait and (ii) the potential of mosses reducing sediment discharge and surface runoff while enhancing water percolation depends on differences in their structural and functional traits. By exploring these hypotheses, this study contributes to the understanding of the functional role of mosses in soil–water interactions, thus having implications for ecological restoration strategies.

## Methodology

### Study site

The study site lies within the Schönbuch Nature Park in southwest Germany, which is located north of Tübingen in Baden-Württemberg (48.543490° N, 9.035727° E). The soil at the study site originates from the geological formation of Angulatensandstein (Lower Jurassic series), which is overlain by periglacial layers that also contain loess (LGRB 2022). The soil type is a Haplic Vertisol according to the IUSS Working Group WRB (2022) and the humus form type is a typical L Mull with the horizontal sequence Ol/Ah according to the German soil classification (Boden 2024). The forest stand consists primarily of conifers, especially silver fir (*Abies alba*) and Scots pine (*Pinus sylvestris*), and a few individual deciduous trees, mainly common beech (*Fagus sylvatica*). The forest floor is widely covered with moss, dominated by the moss species *Thuidium tamariscinum* (Hedw.) Schimp. (approx. 90%), with *Rhytidiadelphus loreus* (Hedw.) Warnst. and *Hylocomium splendens* (Hedw.) Schimp. occuring in larger patches, and *Plagiomnium undulatum* (Hedw.) T.J.Kop., *Hypnum jutlandicum* Hedw., *Eurhynchium angustirete* (Broth.) T.J.Kop., and *Eurhynchium striatum* (Hedw.) Schimp. appearing sporadically.

### Experimental design

To collect undisturbed topsoil samples, we used metal sampling frames with sharp cutting edges measuring 30 cm × 40 cm and subsequently transferred them into infiltration boxes. These boxes, with dimensions of 30 cm × 40 cm and a depth of 15 cm, contain a substructure of perforated metal positioned 6.5 cm below the surface, where we placed the soil monoliths (Gall et al. 2025a). We positioned the soil monoliths in such a way that the mineral soil surface is aligned with the channel, while the mosses protrude above the surface. This monolith sampling preserves the soil and moss structure, enabling ex situ experiments to closely mimic *in situ* conditions. However, ex situ experiments allow full control over factors such as slope and wind. In total, we collected four replicates close to each other per treatment. This combines the advantages of indoor and outdoor experiments in soil hydrology and soil erosion (Ramler and Strauss 2023). For each replicate, we also collected soil samples for the laboratory analysis of basic soil properties.

**Figure 1:**
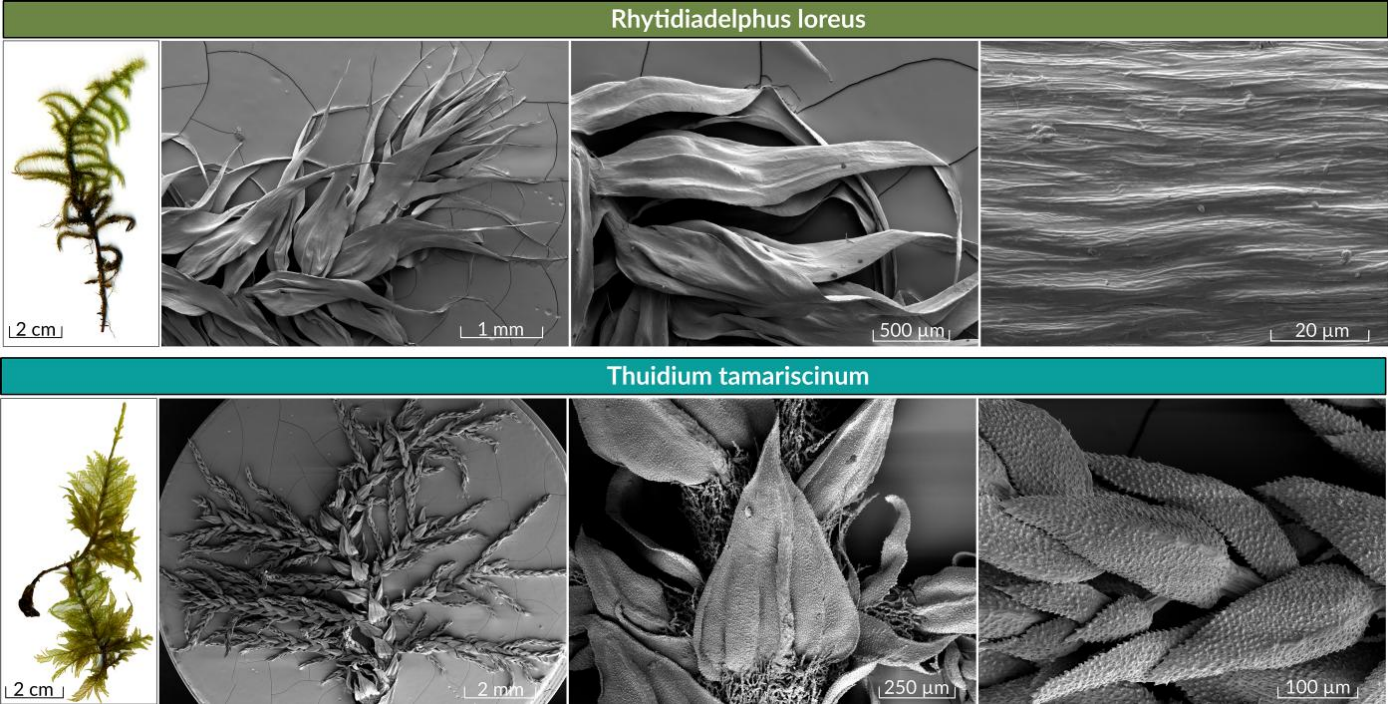
Macro- and micromorphology of the two moss species shown by scanner and scanning electron microscopy (SEM) images. The upper row shows *Rhytidiadelphus loreus*: (from left to right) scanner image of one shoot followed by three SEM images of a shoot tip, leaves, and a leaf surface. The lower row shows *Thuidium tamariscinum*: (from left to right) scanner image of one shoot followed by three SEM images of a shoot with lateral branches, an abaxial leaf surface with shoot leaves, and branch leaves.

**Figure 2:**
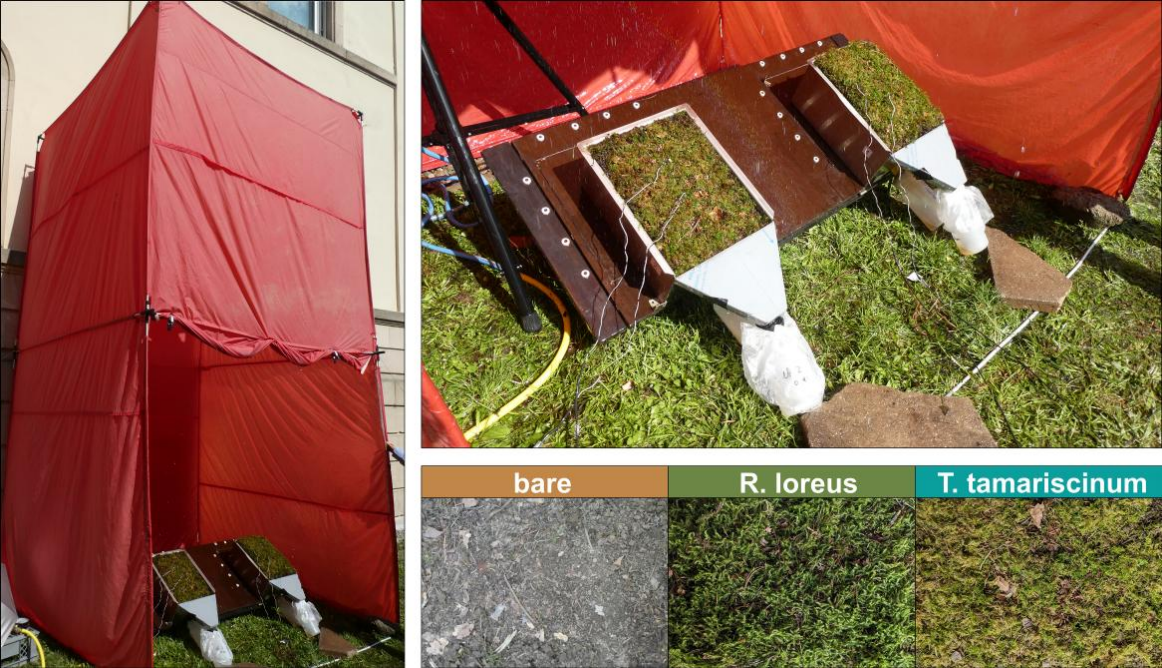
Overview of the experimental setup with the Tübingen rainfall simulator in its protective tent (left), two moss treatments during the rainfall simulation on a table with a 20° slope (upper right), and the three studied treatments (lower right).

Overall, we collected three treatments and named them as follows: bare, *R. loreus* and *T. tamariscinum* (Fig. 2). The bare treatment contains the mineral topsoil with accumulated organic carbon (Ah) and incorporated leaf and occasional needle litter residues without a moss cover. As part of the treatment preparation, we removed the leaf and needle litter that covered the soil surface (Ol horizon) to obtain an uncovered soil surface as a reference sample. Sampling for the bare treatments took place near the two moss treatments in a small common beech stand. The *T. tamariscinum* treatment consists of pure *Thuidium tamariscinum*, while the *R. loreus* treatment is comprised of pure *Rhytidiadelphus loreus*. In both cases, the mosses were naturally growing on the humus layer with 100% coverage, and the moss cover was collected together with the underlying humus and topsoil as an intact, undisturbed sample. *T. tamariscinum* produces rhizoids on parts of the stem that contact the humus, while *R. loreus* does not form rhizoids in mature plants. Both moss species are native to southwest Germany (Nebel et al. 2001), and they differ importantly in their morphology (Fig. 1). *R. loreus* grows as tall turfs with upright and robust, little-branched shoots, while *T. tamariscinum* grows in wefts with a more complex, tripinnate, fern-like structure, creating branches of second and third order (Mägdefrau 1982, Bates 1998, Atherton et al. 2010). The differences in morphology are also apparent in the leaves: While the leaves of *R. loreus* are approximately the same size on the stems and branches, the leaves of *T. tamariscinum* are narrower and shorter with increasing branching order (Atherton et al. 2010).

We carried out rainfall simulations with all studied treatments to measure surface runoff, sediment discharge, and water percolation. For this purpose, we used the Tübingen rainfall simulator with a drop falling height of 3.5 m (Iserloh et al. 2013). Rainfall was simulated with a mean rainfall intensity of 45 mm h^−1^ for 30 minutes, which is classified as a heavy rainfall event according to the German Weather Service (DWD Climate Data Center 2020). In each run, we placed two infiltration boxes on a table with a 20° slope. High rainfall intensity and steep slope angles were chosen to generate high sediment discharge, thereby highlighting the differences between the treatments more clearly. We collected surface runoff, sediment, and percolated water in 1 l sample bottles (Fig. 2).

### Laboratory analysis & measurement of moss traits

Following the rainfall simulations, we determined the amount of surface runoff and percolated water using the measuring scale of the sample bottles. The surface runoff samples were then evaporated at 40°C in a compartment drier to weigh the eroded sediment. For the topsoil samples, we determined the following basic soil properties in the laboratory: Grain size distribution with an X-ray particle size analyser (Sedigraph III 5120, Micromeritics, Norcross, GA, USA), soil pH in 0.01 M CaCl_2_ solution with a pH-meter and Sentix 81 electrodes (WTW, Weilheim, Germany), soil organic carbon (SOC) and total nitrogen with an elemental analyser (Vario EL III, Elementar Analysensysteme GmbH, Hanau, Germany). We applied the water drop penetration time test according to Dekker et al. (2009) and did not observe water repellency.

Moss samples of *R. loreus* and *T. tamariscinum* were collected randomly in the immediate vicinity of the collection sites for the three treatments described above, to measure structural and functional traits. The specimens were determined with the help of a Zeiss Discovery V8 dissecting microscope and a Leitz SM-Lux compound microscope. We determined the following structural traits for both moss species: shoot length, branch length, length of a single component (sum of stem length and length of attached branches), shoot density (number of shoots per sample area), cushion height, ramification (number of branches per shoot), leaf frequency (number of leaves per 1 cm of shoot/branch length), and leaf area. To do so, we first created four replicates of each moss species by cutting the intact moss cushions into circular samples with a horizontal-ground surface area of 5.5 cm in diameter (sample area). We measured cushion height by placing a ruler on the intact moss cushions and the maximum height was given. In the next step, we dissembled these moss samples into single moss shoots, which we placed on a high-definition flatbed scanner (Epson Perfection 4490 Photo, Suwa, Japan). Since the shoots of *T. tamariscinum* had dried up very densely when scanning took place, we immersed them in water for a few minutes before placing them on the scanner, to allow for flat placement and proper scanning. These scans were used to measure shoot and branch length using ImageJ version 1.54g (Schneider et al. 2012). Shoots from the apical meristem to the tip were defined as the longest piece of the above-ground plant available. Ramification was calculated as ramification index:


$$R_{i}=\frac{n_{branches}}{n_{shoots}}$$

with *n* measurements per sample.

For measurement of leaf traits, all leaves along 1 cm of shoot were removed and fixed in gelatine on microscopic slides. Per sample, three replicates were prepared. In *R. loreus*, shoot pieces were selected to be as long as possible; if necessary, shoots and adjacent branches were used. For *T. tamariscinum*, in every case adjacent plant parts adding up to 1 cm in length had to be combined since no long enough pieces without branching were available. Here, samples always consisted of leaves from different order branches. As with length measurements, microscopic slides were then scanned to determine the area and number of leaves per replicate in ImageJ.

For scanning electron microscopy (SEM), exemplary thalli of each species were taken from fresh collected specimen. The moss plants were soaked in tap water and mounted moist on aluminium stubs using conductive carbon cement. After drying at room temperature, they were sputter-coated using a Leica EM ACE200 with palladium and a coating thickness of 12 nm. SEM was performed with a Cambridge Stereoscan 200 at 15 kV.

Additionally, we determined the maximum water storage capacity (WSC) as a functional trait for each moss species following the procedure in Thielen et al. (2021). Therefore, we used intact moss cushions, cutting them into circular samples with a horizontal ground surface area of 10 cm in diameter, and creating five replicates per species. We placed these moss samples between two soil analysis sieves (mesh size of 63 µm on the bottom and 630 µm on the top) and immersed them in deionized water for 5 minutes. After we removed them from the water, the sieve was held askew for 1 minute letting access water run out before we placed them on a table to drip off for another 2 minutes. Then, moss samples were removed from the sieve and placed on plastic petri dishes to be weighed (Mettler Toledo MS603S, Columbus, USA). Subsequently, we dried the moss samples at 30°C for 42.5 hours (Memmert UFE600, Schwabach, Germany) and weighed them to obtain the dry weight. WSC was then calculated as follows:


$$WSC=\frac{wet\;\;weight-dry\;\;weight}{dry\;\;weight}.$$

### Data analysis

We carried out the data analysis using the R software version 4.4.1 (R Core Team 2024). Prior to all statistical hypotheses tests, we inspected residuals of all measured parameters for normality and variance homogeneity using Shapiro–Wilk test and Levene's test. There was neither a normal distribution nor homogeneity of variances for a large part of the data, except for the parameters surface runoff and water percolation. However, we consider our sample size (*n* = 4) as too small to assume normality (Le Boedec 2016), regardless of the produced test results. Therefore, we decided to conduct the following non-parametric test statistic for each measured parameter: In case of comparison of two treatments, we conducted the Wilcoxon rank-sum test, while in case of multiple comparisons, we applied Kruskal–Wallis test in combination with Pairwise Wilcoxon rank-sum test with the *P*-adjustment method ‘Bonferroni–Holm’. To investigate the relationship of moss structural traits to sediment discharge, surface runoff, and water percolation, we created a correlation matrix using the Spearman correlation coefficient, as a meaningful multiple linear regression analysis was not possible with our small sample size. In all cases, we postulated significant differences at *P* < 0.05. To describe the data in the text, we added the median ± interquartile range for individual treatments. The colours selected for all figures are from the ‘wesanderson’ R package (Karthik et al. 2018), with the exception of Fig. 5, for which we used colours from the ‘RColorBrewer’ R package (Neuwirth 2022).

## Results

### Differences in structural and functional traits between moss species

**Figure 3:**
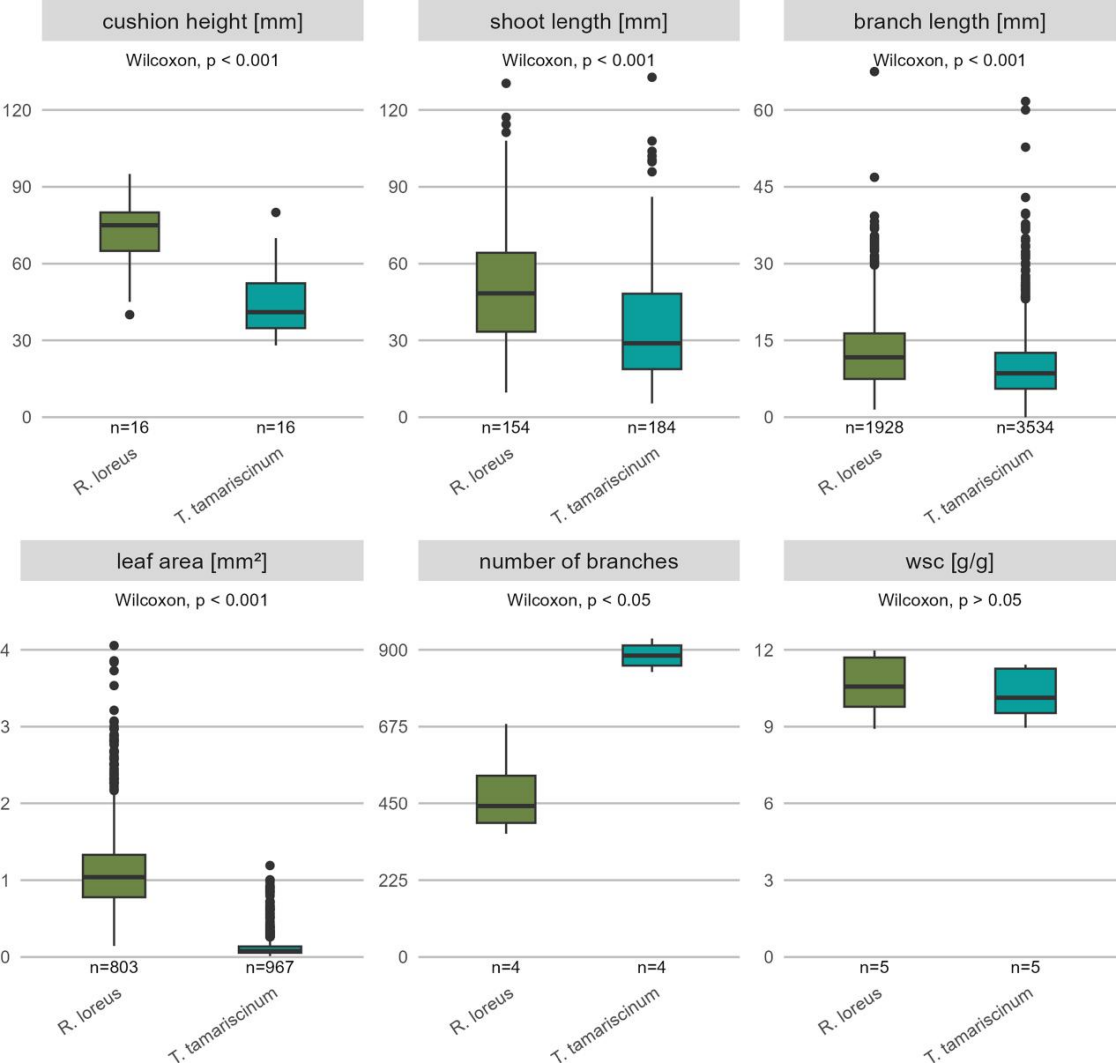
Cushion height, shoot length, branch length, leaf area, number of branches, and maximum water storage capacity (WSC) of the two moss species *Rhytidiadelphus loreus* and *Thuidium tamariscinum*. Lines within the box plots represent median values, while the bottom and top of the box plot show the first and third quartiles, respectively. Whiskers extend up to 1.5 times the interquartile range (IQR) of the data. Outliers are defined as more than 1.5 times the IQR and are displayed as points. The *P* values presented indicate significant differences based on Wilcoxon rank-sum test.

There are significant differences between *T. tamariscinum* and *R. loreus* for cushion height, shoot length, branch length, leaf area, and number of branches (Fig. 3), while shoot density, length of a single component, ramification, and leaf frequency did not significantly differ. *R. loreus* exhibited significantly longer shoots (*R. loreus*: 48.31 ± 30.91 mm; *T. tamariscinum*: 28.84 ± 29.44 mm), as well as longer branches (*R. loreus*: 11.68 ± 8.88 mm; *T. tamariscinum*: 8.56 ± 7.00 mm), which also leads to a higher cushion height. Similarly, leaf area was notably larger in *R. loreus* (*R. loreus*: 1.04 ± 0.55 mm^2^; *T. tamariscinum*: 0.08 ± 0.08 mm^2^), whereas *T. tamarscinum* exhibited a higher number of branches (*T. tamariscinum*: 883 ± 59; *R. loreus*: 442 ± 139). Surprisingly, water storage capacity did not differ between the two moss species despite their differences in structural traits.

### Effect of moss species and their structural and functional traits on sediment discharge, surface runoff, and water percolation

**Figure 4:**
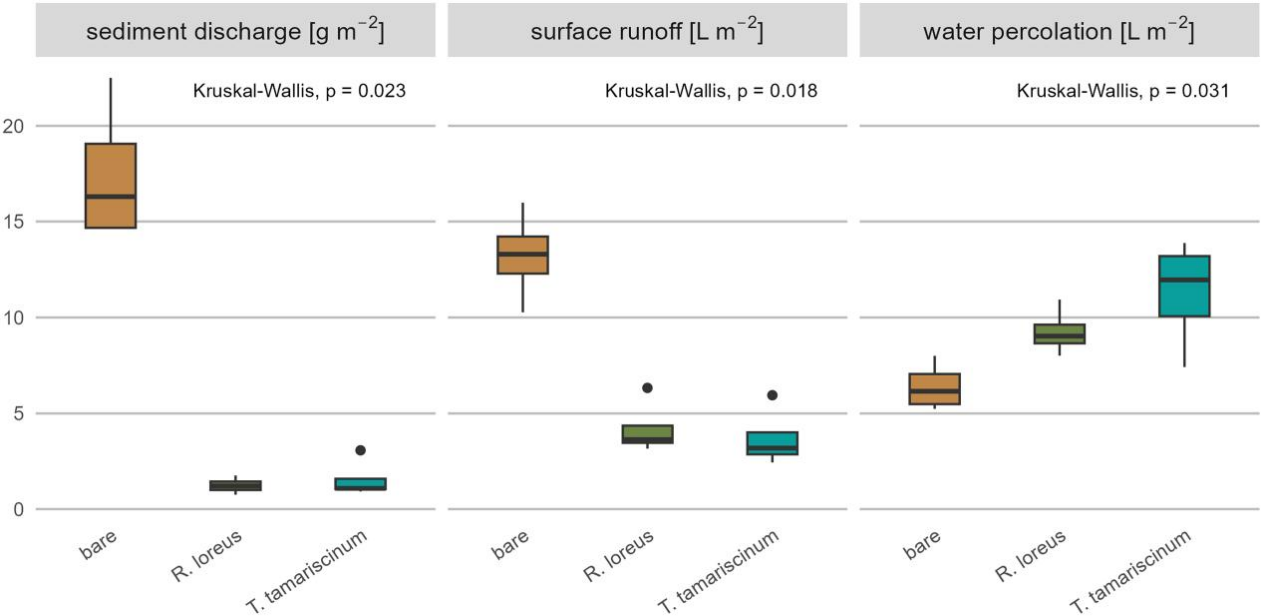
Sediment discharge, surface runoff, and water percolation of the three treatments (bare soil, *Rhytidiadelphus loreus*, and *Thuidium tamariscinum*) measured during rainfall simulations (*n* = 4). Lines within the box plots represent median values, while the bottom and top of the box plot show the first and third quartiles, respectively. Whiskers extend up to 1.5 times the interquartile range (IQR) of the data. Outliers are defined as more than 1.5 times the IQR and are displayed as points. The *P* values presented indicate significant differences based on Kruskal–Wallis test.

The bare treatment produced significantly more sediment discharge (16.30 ± 4.40 g m^−2^) than *R. loreus* (1.21 ± 0.44 g m^−2^, *P* < 0.05) and *T. tamariscinum* (1.08 ± 0.54 g m^−2^, *P* < 0.05). Similarly, surface runoff was significantly higher in the bare treatment (13.30 ± 1.93 L m^−2^) compared to *R. loreus* (3.64 ± 0.90 L m^−2^, *P* < 0.05) and *T. tamariscinum* (3.20 ± 1.14 L m^−2^, *P* < 0.05). However, no significant differences were found between the moss species for either sediment discharge or surface runoff. Water percolation was slightly higher in the moss treatments (*R. loreus:* 9.03 ± 0.97 L m^−2^; *T. tamariscinum:* 11.96 ± 3.13 L m^−2^) than in the bare treatment (6.16 ± 1.57 L m^−2^), but differences were not significant. These results indicate that mosses reduce sediment discharge and surface runoff compared to bare soil, while water percolation is not affected (Fig. 4).

**Figure 5:**
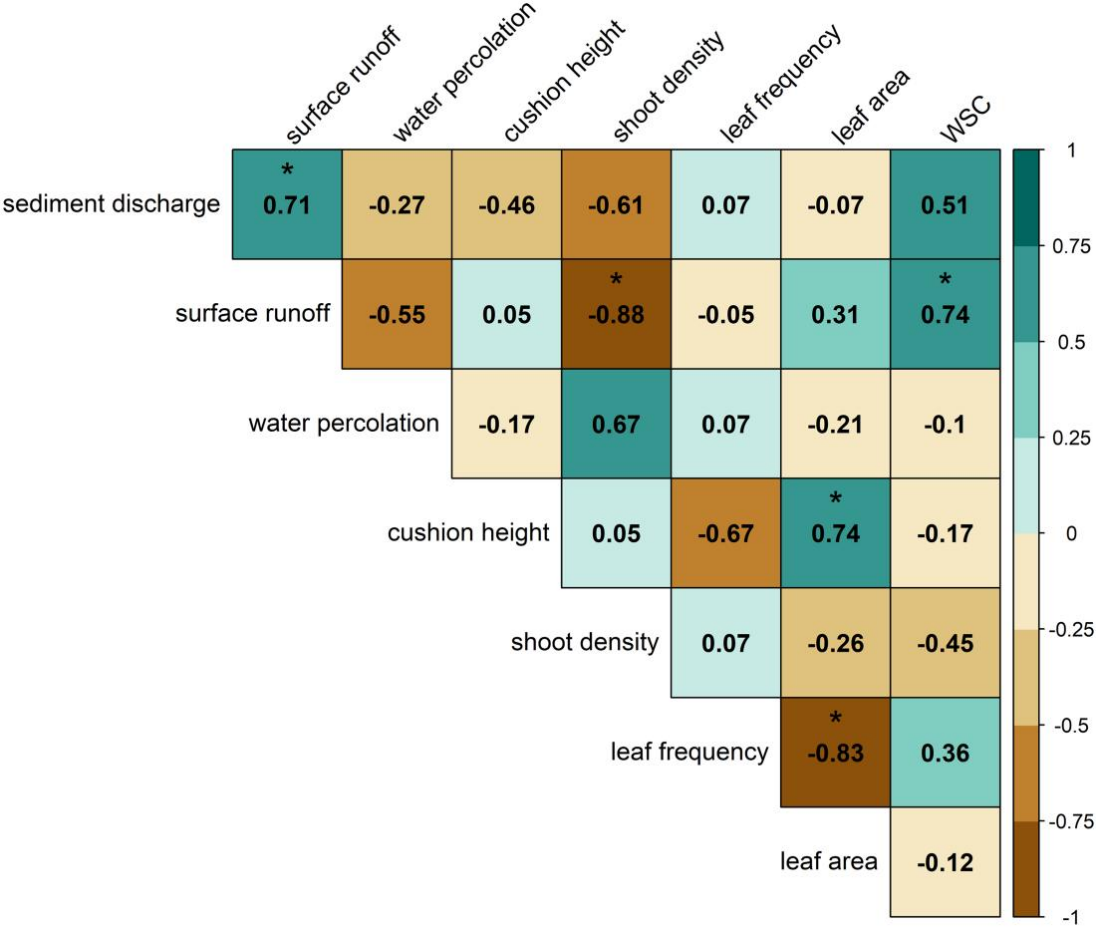
Correlation matrix that shows Spearman correlation coefficients calculated from all moss samples combined (*n* = 8), with asterisks marking statistically significant correlations (*P* < 0.05). Turquoise colours indicate positive, brown colours negative correlations. The following variables are displayed: sediment discharge (g m^−2^), surface runoff (L m^−2^), water percolation (L m^−2^), cushion height (cm), shoot density (*n* cm^−2^), leaf frequency (cm^−1^), and leaf area (mm^2^), WSC = maximum water storage capacity (g g^−1^).

To explore potential relationships between structural and functional traits measured in the moss species and sediment discharge, surface runoff as well as water percolation, a correlation matrix using the Spearman correlation coefficient was generated (Fig. 5). Although more structural moss traits were considered overall, only those that correlate significantly with each other or with sediment discharge, surface runoff or water percolation are shown in Fig. 5. The strong negative correlation between the shoot density and surface runoff should be emphasized here, while there is a positive correlation between surface runoff and the maximum water storage capacity. Furthermore, there is a strong negative correlation between leaf area and leaf frequency, and a positive correlation between cushion height and leaf area.

## Discussion

### Differences in structural and functional traits between moss species

Our hypothesis that *R. loreus* and *T. tamariscinum* differ in their structural traits is partially supported by our data. While *R. loreus* forms higher cushions, longer shoots, and branches as well as larger leaves than *T. tamariscinum*, no difference in shoot density, length of a single component, ramification, and leaf frequency was observed. However, *T. tamariscinum* forms significantly more branches than *R. loreus*. Unexpectedly, the two species did not differ in leaf frequency, although *T. tamariscinum* is known to have an exceptionally fine and complex branching structure of three orders with smaller leaves at increasing branching order (Nebel et al. 2001).

Even more surprising was the observation that, despite the structural differences between *R. loreus* and *T. tamariscinum*, they exhibited similar maximum water storage capacities. Originally, we expected a higher water storage capacity in *T. tamariscinum* due to its above-described complex branching structure and the formation of numerous paraphyllia (Nebel et al. 2001), which increase the surface and thus promote the storage of external water (Hedenäs 2001, Proctor 2008). In addition, before measuring the structural traits, we suspected small leaf sizes with a higher leaf frequency in *T. tamariscinum*, which according to Thielen et al. (2021) correlates positively with water storage capacity. However, for *R. loreus* and *T. tamariscinum* we found no support for this relationship.

One possible explanation for the similar water storage capacities despite different morphological structures may lie in how the leaf structures of the studied species interact with water. In *R. loreus*, larger leaves remain widely spread both under dry and wet conditions, likely allowing faster and more effective wetting of both leaf surfaces. In contrast, *T. tamariscinum* appears finely divided at close range. Still, its first-order lateral branches form larger, contiguous, leaf-like structures when viewed from a distance, reducing open space between branches. This architecture resembles the ‘false leaves’ formed by scale-like foliage in North American conifers such as *Chamaecyparis* and *Thuja*, from which the genus name ‘*Thuidium*’ is derived. In temperate rainforests, such leaf arrangements are thought to enhance evaporation dynamics, and similar structural patterns are observed in other forest-floor mosses like *Plagiomnium affine*, *Eurhynchium* spp., and *Pleurozium*, which tend to have larger leaf blades (Nebel et al. 2001). These resemblances may partly explain similar water storage capacities between *R. loreus* and *T. tamariscinum*.

A further reason for these unexpected results could be that the water storage capacity of mosses is influenced not only by the individual plant morphology but also by the colony structure (Elumeeva et al. 2011, Zajączkowska et al. 2017). While certain structural traits such as small leaf area and high leaf frequency (Thielen et al. 2021), or large leaf widths (Bengtsson et al. 2020) can enhance water storage capacity, their impact can also vary among species, depending on the individual composition of the colony. For example, in several *Sphagnum* species the presence of certain branch types contributed significantly to water retention, with *Sphagnum capillifolium* being an exception (van de Koot et al. 2024). In addition, the variability in the external water storage within one moss species is very high, which is an indication that the structural traits of each individual change considerably between samples of the same species (Volkova et al. 2023).

In summary, it is important to recognize that the water storage capacity is species-specific and likely influenced by a combination of structural traits that interact with each other creating an individual capillary conducting system for external water (Proctor 1982). Therefore, even if *T. tamariscinum* has more branching and forms numerous paraphyllia, other factors such as leaf structure, cell types, and colony density could compensate for the expected increase in water storage capacity.

### Effect of moss species and their structural and functional traits on sediment discharge, surface runoff, and water percolation

Overall, our hypothesis that mosses reduce sediment discharge and surface runoff is supported by our results. Both moss species showed a similar mitigating effect on sediment discharge and surface runoff, and there was no significant effect of moss cover on water percolation regardless of the species. Several studies have measured the reducing effect of mosses on surface runoff and soil erosion (Juan et al. 2023, Gall et al. 2025a), whereby indications also exist that there are species-specific differences concerning the reduction of surface runoff (Tu et al. 2022). Now we could assume that the lack of difference in water storage capacity between *R. loreus* and *T. tamariscinum* is also responsible for the fact that we could not measure any differences in the influence on surface runoff and sediment discharge between the moss species in our case. While water storage capacity is an important functional trait, it is certainly not the only decisive factor that determines a moss's effectiveness in erosion control or runoff mitigation. Other moss structural traits, such as shoot density, leaf frequency, life form, the interconnectedness of shoots and branches, or the ability to form rhizoids are assumed to also play an important role as well.

For example, the correlation analysis shows a significant negative correlation between shoot density and surface runoff, which highlights the important role of the colony structure of mosses on hydrological processes. At the same time, surface runoff is positively correlated with the maximum water storage capacity, which may appear contradictory at first glance. However, this relationship likely reflects fundamental differences between laboratory measurements and field conditions. In the laboratory, maximum water storage capacity is determined by immersing moss samples in water, allowing for complete saturation over extended periods (Mägdefrau and Wutz 1951, Wang and Bader 2018). In this context, Thielen et al. (2021) demonstrated that mosses exhibit different maximum water storage capacities when immersed compared to field conditions, where they do not reach their maximum water storage capacity, instead transmitting water to the underlying soil surface. During rainfall events, water falls onto the moss cushion from above and is not distributed uniformly across the moss surface but is initially retained through lateral water transfer within the cushion. Denser moss cushions promote a greater lateral water transfer (Rice 2012), which may lead to a delay in surface runoff, and an increased downward movement that also supports infiltration into the soil.

In contrast, most of the other investigated structural traits such as cushion height, shoot and branch length, leaf area, or number of branches are not related to sediment discharge, surface runoff, and water percolation. This suggests that, although *R. loreus* and *T. tamariscinum* differ significantly in these traits, such differences do not necessarily translate into corresponding variations in hydrological responses. Nevertheless, these coefficients should be interpreted with caution, as the small overall sample size limits statistical power and does not support species-specific correlation analyses. Moreover, the merging of both species in the correlation analysis may obscure species-specific trends or artificially inflate associations due to interspecies differences. It is therefore important for future studies to select species that differ in certain structural traits and to widen the trait set with higher replication.

## Conclusion

In this study, we investigated and compared structural and functional traits of the two moss species *Rhytidiadelphus loreus* and *Thuidium tamariscinum* and determined their effect on sediment discharge, surface runoff, and water percolation using ex situ rainfall simulations with infiltration boxes. Regarding our two hypotheses, the following conclusions were drawn:

1. Although we detected several structural differences between the two moss species, these differences did not translate into variations in their maximum water storage capacity. This finding is unexpected, particularly given the abundance of fine structural elements in *T. tamariscinum* visible in the SEM images. The underlying causes might be related to trait resemblances and internal leaf-like structures, which need further investigations. Future studies could explore this further by employing 3D imaging of moss cushions to account for their internal pore structure.
2. Both moss species effectively reduced sediment discharge and surface runoff while slightly enhancing water percolation. Although *R. loreus* and *T. tamariscinum* differed in their structural traits, these differences did not lead to differently pronounced hydrological effects. Shoot density emerged as a key structural trait influencing surface runoff, underscoring the importance of moss architecture in soil–water interactions. Future studies should investigate these relationships across a wider range of moss growth forms and under natural field conditions to better understand the mechanisms linking moss structure and hydrological function.

## Data Availability

The dataset compiled and analysed in this study, as well as the R code for reproducing the figures and statistical analysis, are available on figshare: https://doi.org/10.6084/m9.figshare.31058020 (Gall et al. 2026).
